# Hybrid T-Helper Cells: Stabilizing the Moderate Center in a Polarized System

**DOI:** 10.1371/journal.pbio.1001632

**Published:** 2013-08-20

**Authors:** Sui Huang

**Affiliations:** Institute for Systems Biology, Seattle, Washington, United States of America

## Abstract

Polarization of cell phenotypes, a common strategy to achieve cell type diversity in metazoa, results from binary cell-fate decisions in the branching pedigree of development. Such “either-or” fate decisions are controlled by two opposing cell fate-determining transcription factors. Each of the two distinct “master regulators” promotes differentiation of its respective sister lineage. But they also suppress one other, leading to their mutually exclusive expression in the two ensuing lineages. Thus, promiscuous coexistence of the antagonist regulators in the same cell, the hallmark of the common “undecided” progenitor of two sister lineages, is considered unstable. This antagonism ensures robust polarization into two discretely distinct cell types. But now the immune system's T-helper (Th) cells and their two canonical subtypes, Th1 and Th2 cells, tell a different story, as revealed in three papers recently published in *PLOS Biology*. The intermediate state that co-expresses the two opposing master regulators of the Th1 and Th2 subtypes, T-bet and Gata3, is highly stable and is not necessarily an undecided precursor. Instead, the Th1/Th2 hybrid cell is a robust new type with properties of both Th1 and Th2 cells. These hybrid cells are functionally active and possess the benefit of moderation: self-limitation of effector T cell function to prevent excessive inflammation, a permanent risk in host defense that can cause tissue damage or autoimmunity. Gene regulatory network analysis suggests that stabilization of the intermediate center in a polarizing system can be achieved by minor tweaking of the architecture of the mutual suppression gene circuit, and thus is a design option readily available to evolution.

The diversity of cell types in the metazoan body arises through a hierarchical cascade of binary branching in the cells' developmental path [1]. Starting in the omnipotent fertilized egg cell, which faces the first “either-or” choice between the extra-embryonic and the inner cell mass lineage [2] (Figure 1A), such binary branching of lineages is seen throughout the development of virtually all tissues.

**Figure 1:**
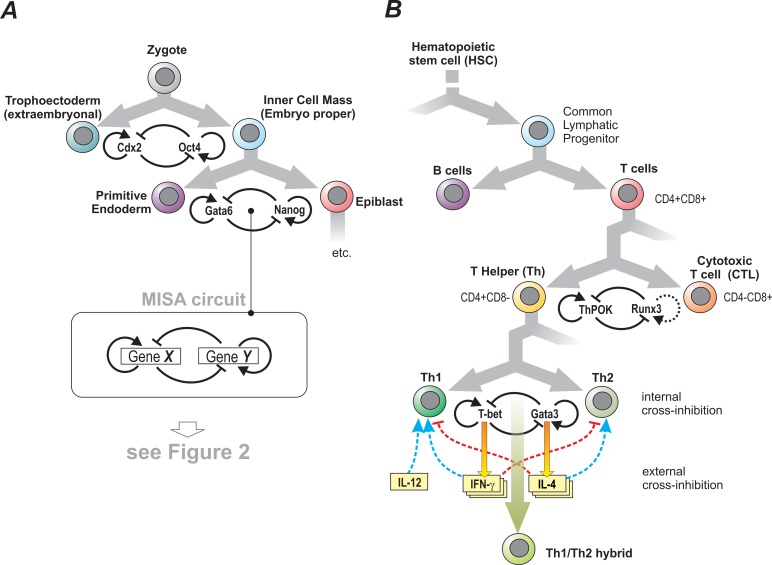
Binary cell-fate decisions in development. Examples of polarization of cell phenotypes at developmental branch points, for the first cell-fate decision in the zygote (A) and for the lymphoid lineages (B). Each binary lineage branching is typically governed by a toggle switch, in which the fate-determining transcription factors often also auto-activate, giving rise to the mutual-inhibition/self-activation (MISA) circuit. In reality these circuits are interconnected to large gene regulatory networks wherein some factors are reused at more than one level of the branching hierarchy. In the Th1–Th2 branching, cross-inhibition between the two lineages as well as self-activation is also mediated by well-characterized external interactions, embodied by the two lineage-characterizing cytokines IFNγ and IL-4 that are also involved in cell proliferation control.

In the adaptive immune system, the common lymphoid progenitor (CLP) has two major lineage options, B lymphocytes and T lymphocytes (Figure 1B). T cells further split into cytotoxic T cells (CTL), identified by the CD8 cell surface marker, and T-helper (Th) cells, which express CD4 instead. This reciprocal surface marker expression is exploited by biologists for the physical separation of these two functionally distinct types of T cells. Not surprisingly, among the highly versatile Th cells a further binary and functionally significant subdivision was discovered in 1986 by Mosmann and Coffman [3],[4]; based on cytokine expression profiles, one can distinguish between Th1 and Th2 cells, which are, roughly speaking, in charge of complementary aspects of host defense [5],[6]. While other Th lineages are now distinguished, the Th1–Th2 dualism results from a tightly controlled “either-or” decision, which is critical because mounting an inappropriate or excessive response against a pathogen can result either in blunted immune defense or in autoimmunity.

Binary branching of cell fates that polarizes the cell phenotype constitutes a natural dichotomy, i.e., the two alternative options are disjoint and mutually exclusive (Figure 1A). How does this polarization arise? Why is black or white prevalent but gray so rare? Conrad Waddington already noted that “intermediates” between discrete phenotypes are rare [7]. But now, in defiance of the ubiquity of such natural polarization of cell lineages, three groups report the existence of a gray-zone “hybrid” Th1/Th2 state that has features of both Th1 and Th2 cells and, importantly, is very stable. Moreover, it does not appear to represent the common metastable precursor, and has a distinct biological function [8]–[10]. How is the polarization overcome to produce such a stable intermediate?

Since one cannot understand “gray” without a preexisting, internalized notion of “black” and “white,” let us take a step back and examine how phenotype polarization so readily and reliably arises in the first place. The emergence of two stable states within one system is one of the first cases of the use of nonlinear dynamical systems theory [11] to predict cell-fate control by a molecular network [12]. This example has now been elevated to a classical paradigm, a gene circuit popularly known as the “toggle switch” [13] (see Figure 2A). Thus a gene circuit consisting of two mutually repressing genes *X* and *Y* can toggle between these two steady states that are stable (attractors and valleys in Figure 2)—***A*** (where *X* is highly expressed and *Y* is suppressed, *X*>>*Y*) and ***B*** (with the reciprocal pattern, *X*<<*Y*). The mathematical description (ordinary differential equations [11]) that maps the mutual repression circuit into such bistable behavior dictates that the intermediate state ***C*** (*X* = *Y*), although a steady state, is unstable (hilltop in Figure 2A).

**Figure 2:**
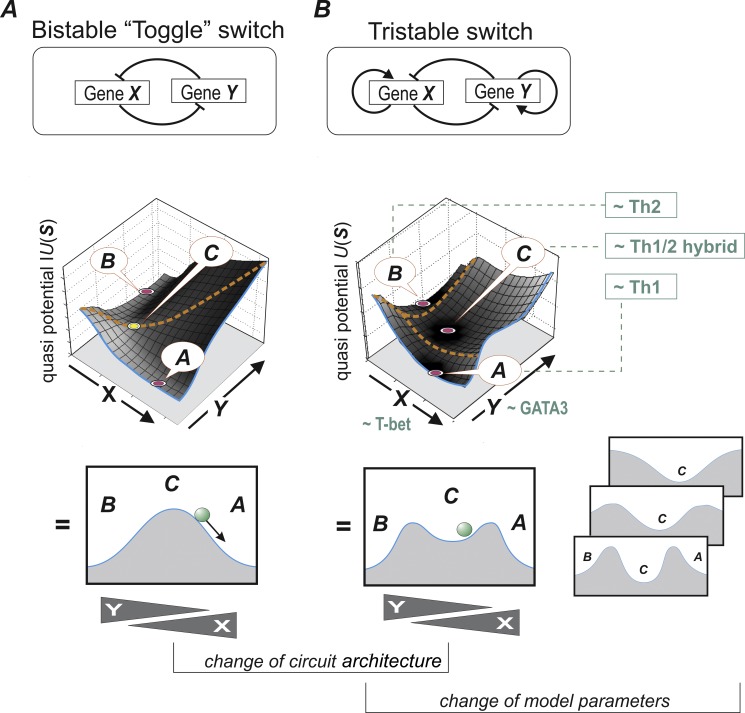
From gene circuit architecture to cell-fate behavior. The theory of dynamical systems predicts the repertoire of cell behavior. The dynamics can be precisely visualized as a “quasi-potential landscape” in which each position represents a state ***S*** (an expression configuration of the two genes *X*, *Y*). The bottom panels show a cross section of the landscape along a diagonal that cuts through the attractors. Here, *steady states* are represented by “flat” regions that experience no driving force. *Stable* steady states (attractor states) are the lowest point in valleys (potential wells) and *unstable* steady states are “hilltops.” Orange dashed lines depict attractor boundaries. The quasi-potential or elevation *U*(***S***) at each ***S*** reflects the “relative stability,” in terms of the probability for state transitions (represented by the height of uphill climb needed to exit an attractor) [16]. The dynamical behavior (manifest in the shape of landscape) is determined by the architecture of the circuit and by the strength and modality of the interactions (model parameters) and gene expression noise. The bistable “toggle”-switch circuit (Panel A) has two stable attractor states ***A*** and ***B***, characterized by reciprocal expression of *X* and *Y*, (*X*>>*Y* or *Y*>>*X*, respectively), whereas the hybrid state ***C*** (*X* = *Y*) is by necessity an unstable steady state. In the tristable “MISA” circuit (Panel B), the central state ***C*** is locally stable. Its relative stability depends on, e.g., the strength of the self-activation loops, which may be the basis for its stabilization in the Th1/Th2 hybrid state. The small insets of landscapes depict examples of their modification by changing the parameters of interactions (“parameter space”). Note here that the central hybrid state can also be modeled as a monostable constellation as done by Antebi et al. [10].

In 1948, Max Delbrück first formulated this principle of cross-inhibition in terms of ordinary differential equations to explain differentiation into two states in a two-metabolite system [14]. Monod and Jacob proposed the same concept in the early 1960s for a two-gene regulatory circuit that is essentially today's “toggle switch” (Figure 2A) [15]. The list of pairs of mutually antagonistic cell-fate regulators that both govern binary lineage branching into two “sister lineages” and regulate their respective effector genes is growing [16]. In the Th1–Th2 dichotomy, the two antagonistic transcription factors are T-bet (promoting Th1) and GATA3 (Th2) [6],[17]. But reality is more complex: binary branching decision points are connected to the larger tree of development, and gene circuits are connected to the complex genome-spanning regulatory network [18]. Yet the presence of mutual suppression switches shines through. Systematic genome-wide analyses of transcriptomes in individual cell types have revealed reciprocal expression for many pairs of regulatory factors in sister cell lineages, and many of them indeed form bidirectional interaction circuits [19].

The binary decision between two fate options implies an undecided decider—a status obviously embodied by the common multipotential precursor of the two sister cell lineages. It is intuitive and plausible that this common precursor is in the intermediate state ***C***, which naturally exhibits “promiscuous” co-expression of the two lineage-specific factors (Figure 2), as first proposed by Tariq Enver [20]. Analysis of gene expression patterns of common precursor cells indeed has provided evidence for such multi-lineage promiscuity [21]–[24]. Simultaneous presence of both antagonistic master regulators in the same cell is thus a hallmark of the precursor of the two respective sister lineages [1].

There is one problem with the promiscuous coexistence of the two opposing regulators *X* and *Y*. According to the mathematical model of the bistable toggle switch, the central, promiscuous state ***C*** (*X* = *Y*) is dynamically highly unstable (Figure 2A)—at odds with biological reality where the promiscuous multipotent stem or precursor cells are discernible cell types that can be isolated and thus display some finite degree of stability despite their notorious propensity to “differentiate away” when not kept in their natural stem cell niche. How can the unstable intermediate state be partially stabilized? Here again, the dynamical systems formalism helps us to understand how the structure of a regulatory circuitry maps into its behavioral repertoire. In addition to the mutual repression between the antagonistic transcription factors, in many toggle-switch circuits the regulators are also capable of (indirect or direct) auto-stimulation, e.g., they may bind to and activate their own promoters [1],[12]. Mathematical modeling shows that self-activation can convert the central state from an unstable steady state to a stable steady state, thus creating “tristable” behavior [23],[25],[26].

The existence of stable steady states (attractors) for a given gene circuit architecture depends both on the structure of its “wiring diagram” and on the parameters in the model that represent the strength of the regulatory interactions (Figure 2). Mathematical analysis of such tristable mutual-inhibition+self-activation circuits (hereafter, MISA) suggests that for a wide range of parameter values the central state ***C*** exists as a stable or at least metastable state [23],[25],[26]. Thus (partial) stabilization of the central state is readily achieved. In other words, tristability per se is a robust phenomenon in the space of possible circuit architectures. Moreover, increasing the strength of autoregulation increases the relative stability [16] of the central attractor state ***C*** [23],[27].

Yet, despite metastability, the central hybrid state is not long-lived—just sufficient to support the undecided precursor state. Multipotent cells quickly make decisions if their state is not actively maintained by its niche. This is why the description of a robust, persistent Th1/Th2 hybrid state by three groups is intriguing [8]–[10].

The existence of a hybrid “Th0” phenotype in activated T cells had been suggested early on based on cytokine profiles [28],[29]. But since cytokine profiles are known to exhibit noisy cell-to-cell variation, the Th0 state remained the subject of debate. More recent work has suggested that virus-specific Th2 cells could be “reprogrammed” to T-bet+/GATA3+ double-positive cells that also exhibit Th1 functionality [30] upon challenge in the context of a Th1-promoting viral infection [30]. Now, the direct generation of Th1/Th2 hybrid cells from naïve T cells and their long-term stability is reported in this issue. The cellular coexistence of T-bet and GATA3, which is more consistent than the co-expression of the subtype-defining cytokines, is documented in a number of ways: at the level of mRNA and protein, and at the functional level. Single-cell resolution analysis was used to unambiguously demonstrate that coexistence of Th1 and Th2 characteristics is due to a “true” (cell-intrinsic) Th1/Th2 hybrid phenotype as opposed to resulting from a *mixture* of Th1 and Th2 cells. The new results also confirm the stochastic manner in which individual cells produce the lineage-specific cytokines. Such “irregular” cytokine expression in subsets of cells [31], once attributed to incomplete polarization or unknown subsets, is now placed in the new context of the stable coexistence of their regulators, T-bet and GATA3, and of the well-recognized stochasticity of gene expression.

In the first report, Fang et al. [8] used fluorescent in situ hybridization to quantitate the transcripts of T-bet and GATA3 and demonstrated that under in vitro nonpolarizing Th cell–activating conditions, Th cells co-expressed both transcripts at high levels in individual cells. This departure from the canonical tristability model, where promiscuous expression typically occurs at intermediate levels [23],[25],[26], may be achieved by adjusting the interaction parameters and the circuit diagram.

In the Th1–Th2 dichotomy, the antagonism also takes place at the level of extracellular communication: The Th1 cytokine IFNγ suppresses the production of Th2 cells, and the Th2-secreted cytokine IL4 suppresses generation of Th1 cells [5],[6]. In the second report, Antebi et al. [10] demonstrate that mixed stimulation with different ratios of IFNγ and IL4 can tune the Th1/Th2 cell state across a large continuum of hybrid Th1/Th2 cells, consistent with a broad regime in which the central state (which in their model is in the monostable regime) is stable.

But is there a biological function for the hybrid state? If the previous two reports relied on spontaneous, unbiased, or (ambivalently) biased conditions for Th activation in vitro, in the third report Peine et al. [9] describe the direct generation of a robust hybrid Th1/Th2 state following in vivo challenge with parasites (which typically elicit Th2 responses). Intriguingly, the Th1/Th2 hybrid cells were stable over an extended period of time (and maintained in the memory T cell state), exhibited immune responses characteristic of both Th1- and Th2-mediated inflammation, and they mitigated immunopathology associated with pure Th1 or Th2 response. Peine et al. also showed that the Th1/Th2 hybrid is unlikely a precursor state because it robustly resisted conversion to Th1 or Th2 cells with IFNγ, IFN-α/β, and IL-12, or with IL-4, respectively.

Here the absence of polarization is literally a moderation of extremes, a compromise at the center, which helps to avoid damage from overt immune response mediated by Th1 or Th2 cells. Did selection for moderation promote evolution of a stable central state? Why regulatory pathways are wired the way they are is a profound problem in systems biology and evolution [32]. Although it is tempting to credit natural selection for tinkering with a circuit's wiring diagram to optimally serve its purpose [33],[34], it is more likely that selection uses those network structures that readily emerge from the random cis/trans region shuffling during genome evolution [35]–[37] and that happen to be associated with a desirable functionality. Natural selection would then only deserve credit for the fine-tuning. As mentioned above, simply adding autoactivation to the toggle switch to obtain a MISA circuit stabilizes the intermediate state. There are other variants of the bistable toggle switch that may have a stable, central hybrid state. For instance, circuits in which one of the cross-inhibitory interactions is mediated by miRNA may also create tristability [38]. Moreover, models that consider gene expression noise, known to produce accumulation of cells in states not predicted by the deterministic (noise-free) model (Figure 2) [39], can also explain an intermediate state—even in the absence of self-activation [40]. Other models that consider more molecular details (transcription factor binding/unbinding, translation) can, for certain parameter values, produce multiple, asymmetric intermediate states and even continuously degenerate hybrid states [41],[42]. In the case of the Th1/Th2 hybrids, the external, cytokine-based MISA circuitry mediated by IFNγ and IL4 (Figure 1B) also functions at the level of cell survival and proliferation control, and may thereby contribute to stabilization of a subpopulation of cells in the hybrid state.

If the stable intermediate state is easily generated in minimal networks, why is it not more frequently encountered in developmental dichotomies governed by MISA circuits, instead being limited to transient metastable precursors? Perhaps there is no need for moderation of extremes in the case of progenitor cells that do not have a major biological effector function in the mature tissue—unlike the immune cells' rapidly deployed defense activities that are often double-edged swords. On the contrary, in development the diversification of cell phenotype is the common objective. Hence robust polarization into clear-cut lineages rather than moderation in the gray zone is desired.

## Abbreviations

CLP: common lymphoid progenitor
CTL: cytotoxic T cells
MISA: mutual-inhibition+self-activation
Th: T-helper
